# Predicting acute renal failure in *Bothrops* snakebite patients in a tertiary reference center, Western Brazilian Amazon

**DOI:** 10.1371/journal.pone.0202361

**Published:** 2018-08-17

**Authors:** Eliane Campos Alves, Jacqueline de Almeida Gonçalves Sachett, Vanderson Souza Sampaio, José Diego de Brito Sousa, Sâmella Silva de Oliveira, Elizandra Freitas do Nascimento, Alessandra dos Santos Santos, Iran Mendonça da Silva, Ana Maria Moura da Silva, Fan Hui Wen, Mônica Colombini, Marcus Vinicius Guimarães de Lacerda, Wuelton Marcelo Monteiro, Luiz Carlos de Lima Ferreira

**Affiliations:** 1 Escola Superior de Ciências da Saúde, Universidade do Estado do Amazonas, Manaus, Brazil; 2 Instituto de Pesquisa Clínica Carlos Borborema, Fundação de Medicina Tropical Dr. Heitor Vieira Dourado, Manaus, Brazil; 3 Núcleo de Sistemas de Informação, Fundação de Vigilância em Saúde do Amazonas, Manaus, Brazil; 4 Instituto Butantan, São Paulo, Brazil; 5 Instituto Leônidas e Maria Deane, FIOCRUZ Manaus, Brazil; 6 Departamento de Medicina, Universidade Federal do Amazonas, Manaus, Brazil; University of Sao Paulo Medical School, BRAZIL

## Abstract

Acute Kidney Injury (AKI) is the main systemic complication and cause of death in viperid envenomation. Although there are hypotheses for the development of AKI, the mechanisms involved are still not established. The aim of this study was to evaluate the clinical-laboratorial-epidemiological factors associated with AKI in victims of *Bothrops* sp envenomation. This is an observational study carried out at the Fundação de Medicina Tropical Dr. Heitor Vieira Dourado. AKI was defined according to the guidelines of the Acute Kidney Injury Network (AKIN). Among the 186 patients evaluated, AKI was observed in 24 (12.9%) after 48 hours of admission. Stage I was present in 17 (70.8%) patients, II in 3 (12.5%) and III in 4 (16.7%). Epidemiological characterization showed predominance of men, occurrence in rural areas, aged between 16–60 years, feet as the most affected anatomical region, and time to medical assistance less than 3 hours. Hypertension and diabetes were the comorbidities identified. Most of the accidents were classified as moderate, and clinical manifestations included severe pain, mild edema, local bleeding and headache. Laboratory results showed blood uncoagulability, hypofibrinogenemia, leukocytosis, increase of creatine kinase, and high lactate dehydrogenase levels. Multivariate analysis showed an association with high LDH levels [AOR = 1.01 (95% CI = 1.01–1.01, p<0.002)], local bleeding [AOR = 0.13 (95%CI = 0.027–0.59, p<0.009)], and the presence of comorbidities [AOR = 60.96 (95%CI = 9.69–383.30; p<0.000)]. Herein, laboratory markers such as high LDH levels along with local bleeding and comorbidities may aid in the diagnosis of AKI.

## Introduction

Snakebite envenomation is a neglected public health problem in tropical countries of underdeveloped continents such as Africa, Asia, Latin America and Oceania [1–3]. Estimates suggest that about 1,800,000 cases and 94,000 deaths occur annually [2]. In Brazil, in 2015, data from the SINAN (national reporting platform) reported 15,454 envenomation cases with an estimated lethality rate of 0.4% [4]. In the state of Amazonas, an average of 1,500 snakebites are reported each year, resulting in an incidence rate of 52.8 cases per 100.000 inhabitants per year, where the proportion of lethality is higher than the national rate of 0.6% [5]. In the Amazon, the *Bothrops* genus is responsible for the highest proportion of snakebites [5–10]. The most important species is *Bothrops atrox* (common lancehead, Amazonian jararaca, jararaca and locally as surucucu) usually found in forested and populated areas [11].

The venom of this genus is composed of a complex mixture of peptides and biologically active proteins that induce a wide range of effects [12–14]. Local and/or systemic manifestations occur due to the tissue injury with subsequent inflammatory mediators release and changes in the coagulation and fibrinolytic system [15–19]. Victims of *Bothrops atrox* envenomation frequently present an intense acute inflammatory response [15] with pain, edema, flushing, bleeding, bruising and blistering at the bite site [20,21]. Systemic effects include headache, nausea, vomiting, spontaneous hemorrhage (gingival, nasal, digestive, hematuria, hematemesis), and more rarely disseminated intravascular coagulation and shock [22,23]. Complications occur due to clinical and environmental factors and include necrosis, secondary infection, Acute Kidney Injury (AKI), intracranial hemorrhage, compartment syndrome, amputations [24–27], and even infrequent situations such as *abruptio placentae* in pregnant women [22] and hepatic hematoma[28].

AKI is the most significant systemic complication and cause of death in envenomation by the *Viperidae* family, with *Bothrops* and *Crotalus* genera being the main cause [29–31]. The prevalence ranges from 10–29% [22,25,31,32] and 1.6–38.5% [31,33–37] respectively, depending on the causative species. AKI is 10 times more common in crotalic accidents than in bothropic, but the incidences are similar due to the greater number of accidents caused by the *Bothrops* genus [31,36]. Venom toxins have direct renal action, changing their structure [30,38–40] and physiology [41] due to capillary vulnerability. Moreover, Acute Tubular Necrosis (ATN) is the most common cause of AKI in snakebite envenomation [38,42,43]. Experimental studies associate this damage to factors such as serpent genus and geographic distribution, severity of the accident, time of exposure to venom [38,44], and inoculated amount of venom [30]. The pathogenesis of AKI has not yet been fully elucidated. However, a multifactorial origin is proposed [45] where some triggering factors would act in a combined or isolated manner [22,32,36,46]. Among these factors are hemodynamic disorders with bleeding or fibrin deposits in tubular structures, inflammatory processes, formation of immune complexes, and nephrotoxic action of venom [30,39,41]. The development of AKI following envenomation has been associated with the age of the individual, where children are more prone due to lower body surface area [25,44], age of the snake [47], venom composition and mechanism of action of the toxins [30,31], time elapsed between the accident and antivenom administration [29,32,35,44], and the presence of comorbidities such as diabetes mellitus and hypertension [43] have been associated with AKI in snakebite patients.

Distance to medical facilities becomes a major hindrance in the Amazon since victims need to travel long distances to healthcare services being prone to complications due to geographical peculiarities [26]. This dynamic results in hampered access to healthcare facilities therefore determining the use of traditional medicine as a treatment option [48]. The magnitude of the problem is expressed by underreporting of snakebites and development of complications [49], which can exemplify AKI. Besides, existing publications on the region only describe the frequency of the event, with no clinical studies especially on AKI [5,26,50]. Although studies point to hypotheses for the development of AKI, its mechanisms have not yet been fully established. Thus, the description of the renal injury pathogenesis based on clinical characteristics presented by patients could contribute to the clarification of the development of acute renal injury induced by envenomation. The aim of this study was to evaluate the clinical-laboratorial-epidemiological factors associated with AKI in victims of envenomation by *Bothrops* snakes.

## Materials and methods

### Study design

This is an observational study carried out from August 2014 to August 2016 at the *Fundação de Medicina Tropical Dr*. *Heitor Vieira Dourado* (FMT-HVD), a reference hospital for snakebite treatment in Manaus, capital city of Amazonas, Brazil, and the only institution in this city offering antivenom therapy. This study was approved by the Ethics Review Board of FMT-HVD (approval n° 602.907-0/2013). All participants signed a consent form after the explanation of the study aims. Under 18 years of age, the consent was signed by parents or guardians. The individual in this manuscript has given written informed consent (as outlined in PLOS consent form) to publish these case details.

### Sample size calculation

Sample calculation was based on two studies conducted in the Amazon region, with an expected frequency of AKI after envenomation of 12% [5,51], at an 80% power and 5% of significance level. The minimum of 174 individuals was necessary adjusting by 15% of lost to follow-up.

### Case selection

All patients included in the study were hospitalized due to the snakebite. Eligible patients were those diagnosed with clinical signs of envenomation by *Bothrops* genus. Pregnant women or individuals that underwent previous antivenom therapy in another hospital unit were not included in this study. According to the Brazilian Ministry of Health Guideline [12], envenomation by *Bothrops* species is classified as follows: I) mild envenomation, characterized by changes in clotting time or not, pain and edema involving one to two segments of the affected limb; II) moderate envenomation, with changes in clotting time or not, pain, evident edema involving three or more segments of the affected limb and local or systemic bleeding without hemodynamic repercussion; III) severe envenomation, that may present changes in clotting time, severe pain, severe hardened edema in the limb, severe hemorrhagic conditions with hemodynamic repercussion, compartment syndrome, and AKI. In addition to the signs of envenomation, patients should have at least two serum creatinine assessments, where the first creatinine test was performed prior to the antivenom therapy.

### Case definition of AKI

At least two measurements of creatinine levels were obtained from each patient during the study period. Acute kidney injury was defined according to the Acute Kidney Injury Network (AKIN) Guideline [52], as follows: Stage I, defined by an increase of >0.3 mg/dL or up to 199% of baseline creatinine levels; Stage II, defined by an increase of 200–299%; and Stage III, an increase in more than 300% in baseline creatinine or serum creatinine levels higher than 4.0 mg/dL with an abrupt elevation of at least 0.5 mg/dL. All patients diagnosed with AKI were referred to the institution’s nephrologist. Those classified as stage III AKI underwent renal replacement therapy.

### Clinical and laboratorial parameters

Data collection included sociodemographic features such as sex, age (in years), anatomical region of bite, area of occurrence (rural or urban), work-related bite, time to medical assistance, clinical and laboratory characteristics (at the moment of the antivenom therapy-D0, first day-D1, second day-D2, third day-D3 and seventh day-D7 of hospitalization), including vital signs, severity classification (mild, moderate or severe), local and systemic manifestations, clinical outcome (discharge or death) and presence of comorbiditites declared by the patient.

Laboratory assays included enzyme immunoassay using monoclonal antibody anti-*B*. *atrox* on admission to assess antigenemia [53], whole blood counts, fibrinogen, serum levels of sodium, potassium, urea, creatinine, alanine transaminase (ALT), aspartate transaminase (AST), erythrocyte sedimentation rate (ESR), C-reactive protein (CRP), creatine kinase (CK). Urinalysis and clotting time were also performed.

### Statistical analysis

A database was built using Epi Info 3.5.1^™^ (Center for Disease Control and Prevention, Atlanta, USA) forms fed by two independent investigators. Data analysis was carried out using the statistical package STATA v13 (Stata Corp, College Station, USA). Explanatory variables were grouped into hierarchical blocks [44,54]. The proximal block was composed of laboratory markers at admission, intermediate block by clinical findings at admission, and distal block by demographic and epidemiological variables. Univariate regression analysis was performed for each block individually. Variables with significance level of *p*<0.2 were included in the multivariate analysis per block. All variables with significance level of *p*<0.05 in the multivariate analysis per block were further included in the overall model (all blocks together). Crude Odds Ratio (OR) and Adjusted Odds Ratio (AOR) with their respective confidence intervals (CIs) were calculated for each hierarchical level and for the global model. The precision of the final model was assessed through Hosmer-Lemeshow goodness-of-fit test [30,45]. The significance level was considered for *p*<0.05.

## Results

### Patients’ characterization

**Fig 1:**
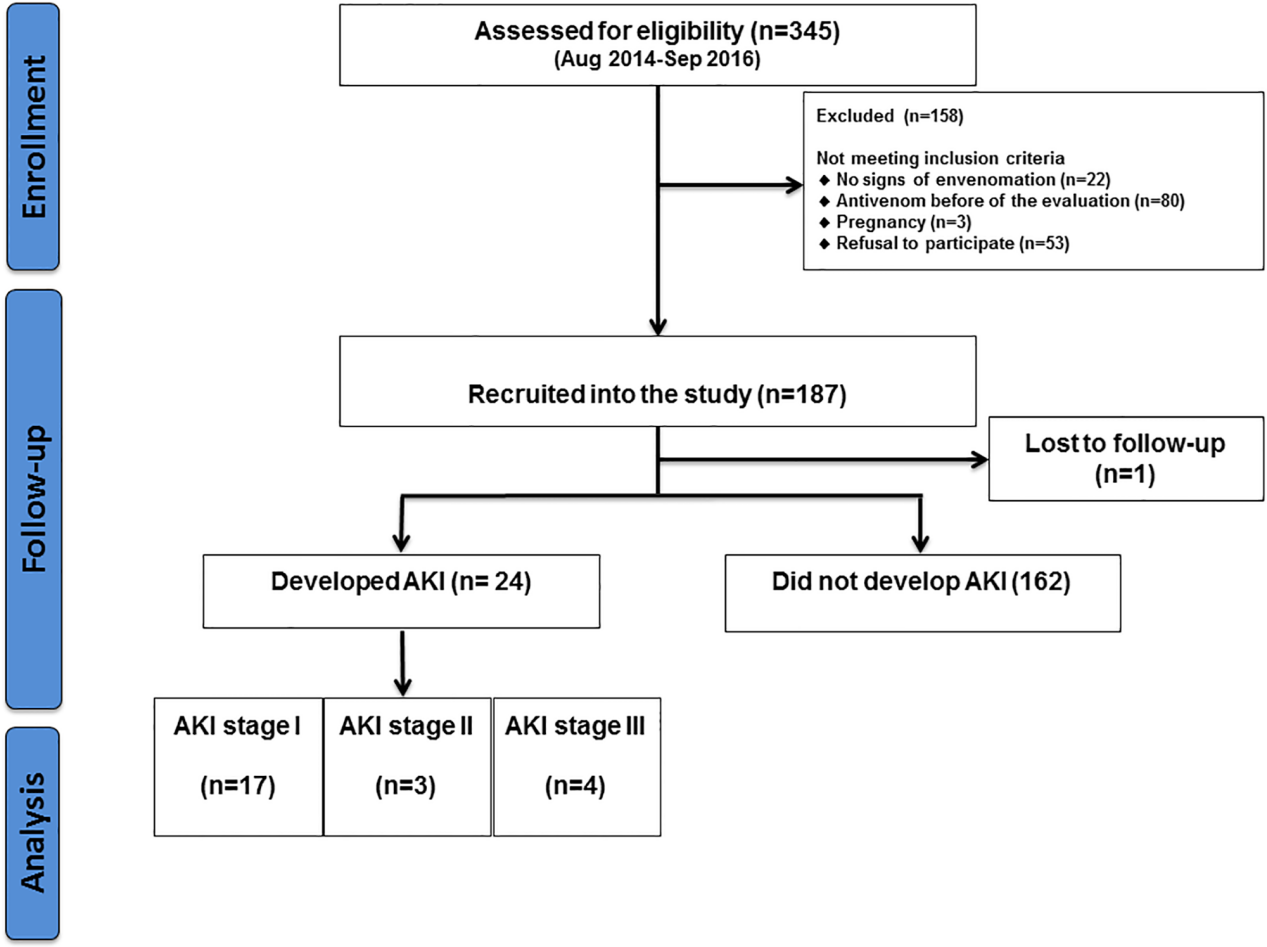
Flowchart of inclusion of patients admitted at FMT-HVD following *Bothrops* snakebite.

A total of 345 patients were assessed for eligibility in the study period. From the total, 187 met the inclusion criteria, with one patient lost to follow-up due to hospital evasion (Fig 1). Enzyme immunoassay confirmed the diagnosis of *Bothrops* sp. envenomation in all patients with complete follow-up.

Patients were mostly male (82.3%), from rural areas (87.1%) and aged between 16–60 years (82.8%). The main anatomical region bitten was foot (66.1%). Most bites were not related to work (59.7%). Time to medical assistance was mostly less than 3 hours (57.5%), the means this time was 4.5 hours and most accidents were moderate (49.0%). Mild and moderate cases were similarly distributed in patients with AKI. A tourniquet on the affected limb was made in 24.7%. No sociodemographic association was found with AKI (Table 1). No patient declared the usage of nonsteroidal anti-inflammatory drugs upon admission. Early adverse reactions to antivenom were observed in 28 patients (16.5%). No late adverse reactions were detected.

**Table 1:** Sociodemographic and clinical characteristics of patients with and without AKI admitted after snakebite.

Characteristics	Without AKI		With AKI		Total
	Number	%	Number	%	
**Sex**					
Male	132	81.5	21	87.5	82.3
Female	30	18.5	3	12.5	17.7
**Area of occurrence**					
Rural	141	87.0	21	87.5	87.1
Urban	21	13.0	3	12.5	12.9
**Age group in years**					
0–15	12	7.41	1	4.1	7.0
16–60	136	83.9	18	75.0	82.8
>60	14	8.6	5	20.8	10.2
**Anatomical region of the injury**					
Upper limbs	1	0.6	1	4.2	1.1
Lower limbs	32	19.8	2	8.3	17.4
Hand	22	13.6	5	20.8	14.5
Foot	107	66.0	16	66.7	66.1
**Work-related accident**					
No	94	58.0	17	70.8	59.7
Yes	68	42.0	7	29.2	40.3
**Time to medical assistance (hours)**					
0–3	93	57.4	14	58.3	57.5
4–6	43	26.5	6	25.0	26.3
7–12	12	7.4	0	0.0	6.5
13–24	14	8.6	4	16.7	9.7
**Previous snakebite**					
No	136	83.9	24	100.0	86.0
Yes	26	16.1	0	0.0	14.0
**Accident classification**					
Mild	70	43.2	10	41.7	43.0
Moderate	81	50.0	10	41.7	49.0
Severe	11	6.8	4	16.6	8.1
**Used oral drugs**					
No	150	97.4	22	91.7	92.5
Yes	4	2.6	2	8.3	3.2
**Used tourniquets**					
No	120	74.1	20	83.3	75.3
Yes	42	25.9	4	16.7	24.7

Clinical characterization showed that the most frequent local manifestations were severe pain (46.2%) and mild edema (48.4%). Among the systemic signs, headache (26.9%), dizziness (14.5%), gingivorrhagia (8.6%), vomiting (7.0%) and nausea (8.1%) were the most common. Comorbities, such as hypertension and diabetes were associated with AKI (p<0.000). Complications namely in the form of necrosis (4.8%) and secondary infection (40.0%) were also identified. No patient presented hypovolemic shock syndrome. Local bleeding was associated with the development of AKI (p<0.001) (Table 2).

**Table 2:** Local/systemic signs, symptoms and complications of the patients with and without AKI admitted to the hospital after snakebite in Manaus, 2014 to 2016.

Characteristics	Without AKI		With AKI		Total
	Number	%	Number	%	
**Local signs**					
**Pain**					
No pain	25	15.4	1	4.2	14.0
Mild	21	13.0	3	12.5	12.9
Moderate	44	27.2	6	25.0	26.9
Severe	72	44.4	14	58.3	46.2
**Swelling**					
Mild (1–2 segments)	79	48.8	11	45.8	48.4
Moderate (3–4 segments)	72	44.4	10	41.7	44.1
Severe (≥ 5 segments)	11	6.8	3	12.5	7.5
**Local Bleeding**					
No	89	54.9	10	41.67	53.2
Yes	73	45.1	14	58.33	46.8
**Systemic**					
Headache	38	23.5	12	50.0	26.9
Dizziness	25	15.4	2	8.3	14.5
Gingival bleeding	13	8.02	3	12.5	8.6
Vomiting	8	4.9	5	20.8	7.0
Nausea	13	8.0	2	8.3	8.1
**Comorbidities**					
**Hypertension**					
Yes	3	1.9	10	41.6	7.0
No	159	98.1	14	58.3	93.0
**Diabetes mellitus**					
Yes	1	0.6	2	8.3	1.6
No	161	99.4	22	91.7	98.4
**Complications**					
Necrosis	8	4.9	1	4.2	4.8
Secondary infection	63	38.9	11	45.8	40.0

Laboratory results revealed that patients with AKI had a slight thrombocytopenia and leukocytosis, increase in creatine kinase, as well as increase in lactate dehydrogenase. Clotting time was uncoagulable in 97.3%. The mean concentration of circulating venom was 44.42 ng/mL (Table 3). Among these parameters, high LDH levels (p<0.001) were associated with the development of AKI. The first blood evaluation occurred in the same time that the patient arrived in the hospital for assistance before administration of the antivenom.

**Table 3:** Laboratory results from patients with and without AKI admitted to the hospital after snakebite in Manaus from 2014 to 2016.

Laboratory tests	Without AKI (n = 162)	With AKI (n = 24)	Total
	Mean (±SD)		
**Antigenemia (ng/dL)**	54.01 (±52.39)	34.83 (±32.55)	44.42 (±42.47)
**Fibrinogen level (mg/dL)**	243.73 (±137.34)	201.54 (±128.73)	222.64 (±133.3)
**Platelets (x10**^**3**^**/mm**^**3**^**)**	230.44 (±75.16)	197.45 (±70.12)	213.95 (±72.64)
**Leukocytes (mm**^**3**^**)**	11.02 (±6.98)	12.85 (±5.41)	11.94 (±6.20)
**Hemoglobin (g/dL)**	13.92 (±1.81)	13.68 (±1.55)	13.80 (±1.68)
**Hematocrit (%)**	40.92 (±5.20)	40.05 (±4.37)	40.49 (±4.79)
**K**^**+**^ **(mmol/L)**	3.89 (±0.37)	4.09 (±0.53)	3.99 (±0.9)
**Na**^**+**^ **(mmol/L)**	139.28 (±5.23)	135.75 (±8.03)	138.52 (±6.63)
**Lactate dehydrogenase (IU/L)**	330.19 (±95.44)	465.13 (±260.34)	397.66 (±177.89)
**TGO/AST (IU/L)**	25.58 (±14.04)	57.88 (±138.64)	41.73 (±76.34)
**TGP/ALT (IU/L)**	23.15 (±20.57)	42.04 (±52.44)	32.59 (±36.51)
**Creatine Kinase (IU/L)**	189.07 (±223.92)	286.66 (±342.69)	237.87 (±283.31)

**Reference values**: Leukocytes: 4.000–10.000/mm^3^; Fibrinogen: 200–400 mg/dL; Platelets: 130,000–400.000/mm^3^; Hemoglobin: 13.0–16.0 g/dL for males and 12.0–14.0 for females; Hematocrit: 40.0–52.0%; Lactate dehydrogenase: 211–423 IU/L; Creatinine: 0.5–1.2 mg/dL for adults and 0.3–1.0 mg/dL for children; Sodium: 135–145 mmol/L; Potassium: 3.6–5.2 mmol/L; Aspartate transaminase (AST): 2–38 IU/L; Alanine transaminase (ALT): 2–44 IU/L; Creatine phosphokinase: 24–190 IU/L.

#### Clinical outcomes and risk factors for AKI

Of the 186 patients in the study, 24 (12.9%) developed AKI after 48 hours of admission, and 162 (87.1%) did not develop such clinical condition. Stage I was observed in 17 (70.8%) patients, stage II in 3 (12.5%), and stage III in 4 (16.7%) patients (Fig 1). All patients who had a diagnosis of AKI were referred to the institution’s nephrologist. Those classified as stage III underwent dialysis. No patients had Chronic Kidney Disease (CKD) at admission ratified by the decrease in creatinine levels during follow-up thus confirming acuteness of disease. There were no deaths.

In the block analysis, considering the proximal variables, after 48 hours of follow-up AKI had significant association with high lactate dehydrogenase levels [AOR = 1.01 (95%CI = 1.01–1.01); *p*<0.001)]. Among the intermediate variables only local bleeding showed association [AOR = 0.13 (95%CI = 0.04–0.45; *p*<0.001)]. As for the distal variables, comorbidities were also significantly associated [AOR = 37.85 (95%CI = 9.32–153.69; *p*<0.001)] (Table 4).

**Table 4:** Variables associated to acute renal failure in patients admitted to the hospital in Manaus from 2014 to 2016.

Variables	Without AKI n (%)	With AKI n (%)	Crude OR (IC 95%)	*p*	Adjusted OR (95%CI)	*p*
**Proximal variables**						
**Antigenemia**						
Detectable	135 (83.3)	18 (75.0)	0.99 (0.97–1.01)	0.301	...	...
Undetectable	27 (16.7)	6 (25.0)	1			
**Blood coagulability**						
Normal	5 (3.1)	0 (0.0)	1			
Uncoagulable	157 (96.9)	24 (100.0)	6.91 (1.98–24.10)	0.002*	3.51 (0.53–23.32)	0.193
**Fibrinogen level**						
Normal	116 (71.6)	20 (83.3)	1			
Abnormal	46 (28.4)	4 (16.7)	0.99 (0.99–1.00)	0.182	...	...
**Thrombocytopenia**						
Yes	157 (96.9)	24 (100.0)	0.99 (0.99–1.00)	0.042*	0.99 (0.98–1.00)	0.396
No	5 (3.1)	0 (0.00)	1			
**Leukocytes**						
Normal	96 (59.3)	10 (41.7)	1			
Abnormal	66 (40.7)	14 (58.3)	1.03 (0.98–1.01)	0.268	...	...
**Hemoglobin**						
Normal	151 (93.2)	23 (95.8)	1			
Abnormal	11 (6.8)	1 (4.2)	0.92 (0.71–1.20)	0.547	...	...
**Hematocrit**						
Normal	161 (99.4)	24 (100.0)	1			
Abnormal	1 (0.6)	0 (0.00)	0.96 (0.88–1.06)	0.432	...	...
**K**^**+**^						
Normal	161 (99.4)	23 (95.8)	1			
Abnormal	1 (0.6)	1 (4.2)	3.48 (1.18–10.23)	0.023*	2.01 (0.36–11.29)	0.424
**Na**^**+**^						
Normal	157 (96.9)	24 (100.0)	1			
Abnormal	5 (3.1)	0 (0.00)	0.91 (0.85–0.98)	0.009*	0.92 (0.84–1.02)	0.140
**LDH (U/L)**						
Normal	135 (83.3)	15 (62.5)	1			
Abnormal	27 (16.7)	9 (37.5)	**1.01 (1.01–1.01)**	0.001*	**1.01 (1.01–1.01)**	0.012*
**TGO/AST (IU/L)**						
Normal	138 (85.2)	17 (70.8)	1			
Abnormal	24 (14.8)	7 (29.2)	1.02 (0.99–1.04)	0.197	0.99 (0.97–1.01)	0.377
**TGP/ALT (IU/L)**						
Normal	127 (78.4)	16 (66.7)	1			
Abnormal	35 (21.6)	8 (33.3)	1.02 (1.00–1.03)	0.015*	1.01 (0.99–1.04)	0.256
**CK- Creatine Kinase**						
Normal	115 (71.0)	13 (54.8)	1			
Abnormal	47 (29.0)	11 (45.8)	1.00 (1.00–1.00)	0.081	1.00 (1.00–1.00)	0.252
**Urine pH level**						
Normal	122 (80.3)	18 (75.0)	1			
Abnormal	30 (19.7)	6 (25.0)	0.58 (0.22–1.49)	0.260	...	...
**Urine protein**						
No	124 (80.5)	17 (73.9)	1			
Yes	28 (19.5)	6 (25.1)	1.56 (0.56–4.32)	0.389	...	...
**Urine hemoglobin**						
No	138 (90.8)	17 (73.9)	1			
Yes	14 (19.2)	6 (25.1)	3.48 (1.18–10.25)	0.024*	1.69 (0.26–10.89)	0.580
**Urine red blood cells**						
No	25 (32.9)	5 (38.5)	1			
Yes	51 (67.1)	8 (61.5)	0.78 (0.23–2.65)	0.695	...	...
**Intermediate variables**						
**Local signs**						
**Pain**						
No pain	25 (15.4)	1 (4.2)	0.71 (0.41–1.25)	0.244	...	...
Mild	21 (13.0)	3 (12.5)	0.66 (0.18–2.42)	0.531	...	...
Moderate	44 (27.2)	6 (25.0)	0.31 (0.07–1.42)	0.133	...	...
Severe	72 (44.4)	14 (58.3)	1.83 (0.18–18.6)	0.608	...	...
**Swelling**						
Mild (1–2 segments)	79 (48.8)	11 (45.8)	1			
Moderate (3–4 segments)	72 (44.4)	10 (41.7)	0.88 (0.33–2.37)	0.806	...	...
Severe (≥5 segments)	11 (6.8)	3 (12.5)	2.11 (0.68–6.49)	0.191	...	...
**Local bleeding**						
Yes	**73 (45.1)**	**14 (58.33)**	**0.13 (0.04–0.45)**	0.001*	**0.13 (0.027–0.59)**	0.009*
No	89 (54.9)	10 (41.67)	1			
**Systemic**						
Dizziness	25 (15.4)	2 (8.3)	0.49 (0.11–2.25)	0.365	...	...
**Complications**						
Secondary infection	63 (38.9)	11 (45.8)	1.96 (0.51–7.61)	0.330	...	...
**Distal variables**						
**Sex**						
Male	132 (81.5)	21 (87.5)	1			
Female	30 (18.5)	3 (12.5)	0.62 (0.17–2.24)	0.475	...	...
**Area of occurrence**						
Rural	141 (87.0)	21 (87.5)	0.97 (0.39–2.41)	0.953	...	...
Urban	21 (13.0)	3 (12.5)	1			
**Age group in years**						
0–15	12 (7.4)	1 (4.2)	1			
16–60	136 (84.0)	18 (75.0)	1.59 (0.19–12.95)	0.666	...	...
>60	14 (8.6)	5 (20.8)	4.29 (0.44–41.95)	0.211	...	...
**Work-related accident**						
No	94 (58.0)	17 (70.8)	0.57 (0.22–1.45)	0.237	...	...
Yes	68 (42.0)	7 (29.2)	1			
**Time to medical assistance (hours)**						
0–3	12 (7.4)	1 (4.2)	1			
4–6	30 (18.5)	4 (16.7)	0.93 (0.33–2.58)	0.884	...	...
7–12	39 (24.1)	3 (12.5)	...	...	...	...
13–24	23 (14.2)	4 (16.7)	1.90 (0.55–6.59)	0.313	...	...
**Accident classification**						
Mild	70 (43.2)	10 (41.7)	1			
Moderate	81 (50.0)	10 (41.7)	0.86 (0.33–2.20)	0.759	...	...
Severe	11 (6.8)	4 (16.6)	2.54 (0.67–9.55)	0.166	...	...
**Used oral drugs**						
No	4 (2.5)	2 (8.3)	1			
Yes	150 (92.6)	22 (91.7)	1.97 (0.81–4.78)	0.130	...	...
**Used tourniquets**						
No	120 (74.1)	20 (83.3)	1			
Yes	42 (25.9)	4 (16.7)	1.79 (0.75–4.26)	0.189	...	...
**Comorbidities**						
No	158 (97.5)	14 (58.3)	1			
Yes	4 (2.47)	10 (41.7)	**37.85 (9.32–153.69)**	<0.001*	**60.96 (9.69–383.30)**	<0.001*

*The significance level was considered for *p*<0.05.

Reference values: Leukocytes: 4.000–10.000/mm^3^; Fibrinogen: 200–400 mg/dL; Platelets: 130,000–400.000/mm^3^; Hemoglobin: 13.0–16.0 g/dL for males and 12.0–14.0 for females; Hematocrit: 40.0–52.0%; Creatine phosphokinase: 24–190 IU/L; Creatine phosphokinase-MB: 2–25 IU/L; Lactate dehydrogenase: 211–423 IU/L; Creatinine: 0.5–1.2 mg/dL for adults and 0.3–1.0 mg/dL for children; Urea: 10–45 mg/dL; Sodium: 135–145 mmol/L; Potassium: 3.6–5.2 mmol/L; Aspartate transaminase: 2–38 IU/L; Alanine transaminase: 2–44 UI/L; Clotting time: 4–10 minutes; pH-urine: 5.5–7.0; Density-urine: 1025–1035.

## Discussion

AKI is the main complication observed in snakebite envenomation [38]. It is estimated that between 1.6 to 38.5% of the victims develop AKI [36]. This wide variation in frequency is due to unclear establishment of the diagnostic criteria.

In this study, AKI was observed in 12.9% of cases, which is similar to previous studies in the Brazilian Amazon by Feitosa et al. and Souza et al. [5,50]. The development of AKI is an unestablished event [45] and, given that, there are proposals for some triggering events of hemodynamic, nephrotoxic and immunological nature [38]. As for circulatory abnormalities, hemorrhagic conditions, hypo or hypertension, vascular changes, intravascular hemolysis and disseminated intravascular coagulation (DIC) are described in the literature [29,43,45].

Vascular disorders occur through hemorrhagic and coagulant action induced by venom components of *Bothrops* snakes that promote endothelial injury by hemorrhagins [13] and consumption of coagulation factors [55]. In South America, this disorder has been caused by snakes of the *Viperidae* family [31,32]. In this study, changes were caused specifically by *Bothrops* snakes, also described in other papers on the Amazon region [5,6,49,56].

In human envenomation, it is has been shown that experimental models with venoms from *B*. *jararaca*, *B*. *moojeni and B*. *alternatus*, induced changes in tubular, glomerular and interstitial structures [39,41,57] indicating the action of venom toxins on the renal parenchyma occurs within a few hours [32]. However, the involvement of immune response in the AKI development still requires more experimental data. Its harmful effect may be caused by deposition of immunocomplexes, IgM and C3 molecules in mesangial areas characterizing glomerulonephritis.

Changes in the renal parenchyma reflect in the clinical presentation, defined and classified according to the International Society of Nephrology’s guideline of clinical practice (KDIGO) [52]. Diagnosis is based on serum creatinine and/or urinary volume and classified into three stages. In this study, stage I was presented by most patients (70.8%), while stage II occurred in 12.5% and stage III, which is indicative of renal replacement therapy (RRT), was present in 16.7% of patients. Due to hospital routine, it was not possible to obtain urinary volume measurement of all patients, which is a limitation of the study. The recommended treatment in these conditions is complementary, and it is necessary to adopt specific initial measures for envenomation and later for complications, supportive measures for hemodynamic disorders and specific actions for AKI [36].

Early and adequate administration of antivenom is crucial. Coagulation factors return to baseline levels between 6 to 24 hours after antivenom therapy [12,21,58]. This fact minimizes venom-induced actions, of circulatory and immunological order, that are related to renal disorder. The management of AKI ranges from the use of antidiuretics to increase urinary flow [59] to renal replacement therapy (RRT) such as peritoneal dialysis[26] and hemodialysis. In India, about 13.5% of the cases developed AKI, of which 48.3% needed dialysis [29]. It is important to note that the most common snakes that cause accidents in India are *Daboia russellii* and *Echis carinatus* that present venoms with hemotoxic and myotoxic actions [30,44].

In this study, there was no significant association between AKI and epidemiological aspects. These were similar to previous studies where it was seen a greater predominance of male individuals, living in rural areas, in productive age group, and having the feet as the main anatomical region reached [7,8,26]. This fact may be explained by the greater exposure to leisure and occupational activities [7].

Regarding clinical aspects, local inflammatory events such as pain and edema were the most commonly identified [5,21,26], venom toxins from *Bothrops* snakes present proteolytic action through inflammatory mediators that act by activating nociceptors and promoting changes in lymphatic vessels [16].

The majority of patients presented blood uncoagulability (97.3%). The pathophysiological mechanism of hemostatic disorders relies on the consumption of coagulation factors and pro-coagulant activation, as well as thrombin-like enzymes [60]. According to the guidelines of the Brazilian Ministry of Health, clotting time is not a diagnostic criterion for the definition of envenomation [12]. Studies indicate 23 to 43.1% of patients who are victims of accidents in the Amazon region present blood uncoagulability, varying from case to case [5,7,26]. In addition, thrombocytopenia is a result of platelet aggregation inhibition by serine proteases [60]. Another parameter related to hemostatic changes induced by the venom was the presence of hemorrhagic conditions, also showed by Athappan et al. [29].

In this study, most patients who developed AKI had comorbidities namely hypertension and diabetes. It is known that such comorbidities lead to increased risk for the development of this condition[51], as a result of long-term damage to target organs such as heart and kidneys. Also, the difference in serum lactate dehydrogenase levels among patients with AKI was significant. This is a marker of cell injury as shown by Castro et al. in experimental models of *B*. *jararaca*-induced tubular injury [39].

In the multivariate analysis, changes in lactate dehydrogenase levels followed by local bleeding and presence of comorbidities, were independently associated with the onset of AKI. This data was shown by Aye et al. where it was observed that the administration of antivenom more than 2 hours from bite, leukocytosis, DIVC, rhabdomyolysis, hyponatremia and microscopic hematuria were factors associated with the clinical picture of AKI [61]. In a retrospective study, independent factors associated with AKI at admission were the presence of hemorrhagic changes and long hospital stay [32].

Thrombocytopenia and liver enzyme release were shown to be statistically significant in those events according to Naqvi et al. [62]. In India, a prospective observational study found that patients who took longer to medical assistance and presented hypotension, albuminuria, anemia, changes in bleeding time, prothrombin time and total bilirubin serum levels, developed AKI [63].

This study had limitations. First, it may underestimate the real prevalence of AKI since only snakebites that took no longer than 24 hours to hospital admission were included in this study. Besides, it was not possible to perform urine volume measurement and calculate creatinine clearance during the study. Imaging tests would also be a good tool to complement diagnosis although not used in this study. For logistical reasons, it was not possible to follow-up participants at the end of the study either personally or by telephone, to identify the chronicity of the case.

## Conclusion

Acute renal failure was an important systemic complication observed in patients envenomed by *Bothrops* snakes. This study may aid in understanding some of the toxin-induced changes in the human body. The associated risk factors for AKI were high lactate dehydrogenase levels followed by local bleeding and the presence of comorbidities, majorly hypertension, suggesting that these laboratory and clinical aspects may aid in the diagnosis, management, clinical evolution and possibly reduce the frequency of these events in those victims. Further prospective studies are needed in order to elucidate the pathogenesis of AKI and to identify the different factors involved.

## Supporting information

S1 Checklist ESTROBE checklist. (DOCX)
